# A Case of Congenital Occlusion of the Vagina, Relieved by Operation

**Published:** 1853-08

**Authors:** Geo. L. Upshur

**Affiliations:** Surgeon to U. S. Marine Hospital, Norfolk; Member of Am. Med. Association; Fellow of Med. Soc. of Virginia, &c., &c.; Norfolk, Va.


*A case of Congenital Occlusion of the Vagina, relieved by operation.* By Geo. L. Upshur, A. M., M. D.; Surgeon to U. S. Marine Hospital, Norfolk; Member of Am. Med. Association; Fellow of Med. Soc. of Virginia, &c., &c.

The subject of this notice was a negro girl, aged 18, the property of Dr. Southall, of Smithfield. She was admitted into the Norfolk Slave Infirmary, October 8th, 1852.

About eighteen months ago, she commenced to have symptoms characteristic of the approaching catamenia, recurring every four weeks with great regularity, and continuing at each return about four days, but without any discharge from the vagina. A round, well defined tumor had gradually appeared above the pubes, becoming larger after every return of the menstrual symptoms, and giving the appearance, when she entered the Infirmary, of one advanced to the fourth month of pregnancy. Eight months ago she began to suffer from distension, and recently the pressure of the tumor upon the rectum and bladder has given her a great deal of pain. There are, also, evident contractions of the uterus, as if it were making a strong effort to get rid of its contents. These symptoms have been so severe and so continuous for the last two months as to unfit her for work, and oblige her to remain almost constantly in the recumbent position. The patient was well grown, and the mamma and external genitals fully developed, the clitoris and labia being of the ordinary size and shape, and the pubis covered with hair.

She stated that she had repeatedly felt the venereal desire, and four times had attempted to consummate the venereal act, but without success. Upon examination I found that the finger would pass into the vagina only about an inch and a-half, being arrested by a firm, thick septum, perfectly smooth, and offering a complete barrier to further ingress. By pressing upon the tumor when the finger was in the vagina, fluctuation could be easily felt. On introducing one finger into the rectum, and a catheter into the bladder, and inclining the former upward, and the latter downward, the finger came in contact with the catheter just behind the terminus of the vagina, nothing appearing to be between them but the coats of the rectum and bladder. The tumor could also be felt from the rectum, sinking low down into the pelvis, and giving decided fluctuation when gently struck.

The introduction of the speculum showed that the vagina was perfectly formed, *as far as it went.* There was no enlargement at its uterine extremity—no *cul-de-sac* formed. It seemed as if nature had lacked for materials to form an entire vagina, but as far as she progressed she had done her work perfectly.

On the 15th of October I performed the operation, in the presence of Drs. Moore, Rose, Robt. Tunstall, Southall, and H. Nash. The bladder and rectum being first evacuated, the patient was placed upon the table, as in the position for lithotomy, except that the hands were not bandaged to the ankles. Chloroform was then administered, and as soon as she became insensible, I introduced the speculum up to the terminus of the vagina, and distended the parts as far as possible. The instrument was held in position by an assistant who, at the same time, made pressure upon the uterine tumor. A second assistant retained a catheter in the bladder, in such a position that I could easily touch it with the forefinger of the left hand introduced into the rectum. Taking the view that in the space bounded by the rectum and bladder, and the vagina and os tincæ, there was nothing but cellular tissue, I desired to have such guides in the operation as would prevent me from wounding any portion of the pelvic viscera, my object being to make a passage up to the os tincæ directly in a line with what would have been the axis of the vagina had it been complete. The tumor above, and the finger and catheter behind and in front, performed the part of such guides.

The operation was commenced by perforating the centre of the obstruction with an ordinary trochar, which was pushed in to the depth of an inch, and the opening widened by a crucial incision with the scalpel, so as to admit the forefinger of my right hand. The speculum was now withdrawn, the finger introduced, and the obstructing tissue *torn* in every direction as far as I could reach. The dense and unyielding nature of the tissue rendered this part of the operation tedious and difficult, and so fatigued the hand that I was glad to accept the aid of Drs. Moore and Tunstall.

The way being opened as far as we could reach with the finger, the speculum was again introduced, passing in this time about four inches, and being arrested apparently about an inch short of the os tincæ. Pressure being still made upon the tumor, I carefully divided layer after layer with the scalpel, until I reached the os tincæ, which was immediately made known by an abundant flow of a fluid resembling tar in color and consistency, and perfectly inodorous.

The hips being lowered so as to promote the discharge, the patient was left for four hours. At the end of this time thirty-two ounces had been discharged, and the flow was still going on *with regular uterine contractions*, which continued for twelve hours, until every particle of the secretion had come away. The whole quantity amounted to forty-eight ounces, or about three pints. The patient having rallied entirely at the end of eight hours, was put to bed, and ordered half grain of sulph. morph., and perfect quiet to be observed.

Oct. 16th.—Patient rested well last night, and feels tolerably comfortable this morning. Pulse 80, soft and regular; tongue natural, and appetite good. Upon examining per vaginam, I could easily feel the uterus. The organ was larger than it is in the unimpregnated state, and the os sufficiently patulous to admit the end of the finger in other respects. There was nothing remarkable about it. The patient did not complain of pain from the introduction of the finger, nor was there the slightest soreness from pressure over the abdomen. In the evening, finding that no urine had been discharged since the operation, I introduced the catheter, and drew off thirty ounces. Ordered milk and bread diet in small quantities.

17th.—Had a restless night, with slight shivering and thirst. Soreness upon pressure over the whole abdomen, but particularly in the hypogastric region. Pulse 110, moderately full; skin dry; tongue covered with white fur; anorexia; thirst; bowels constipated, and vaginal discharge whitish, and beginning to be fetid. Ordered an ounce of sulph. magnes. to be given at once, and the vagina to be syringed with warm water dashed with vinegar, three times a day. Warm fomentations to the abdomen.

18th.—Less soreness than yesterday over the abdomen. Vagina very tender to the touch ; discharge feculent ; bowels moved once ; complains bitterly when the syringe is introduced. Continue treatment.

19th.—General symptoms as yesterday. Vagina hot and tumefied ; discharge very fetid ; great pain upon introducing the finger ; urine drawn off night and morning since the day after the operation. Continue treatment.

20th.—Parts very tender, and very little discharge from them. Administered chloroform, and made a minute examination with the speculum. Vaginal surface intensely red, swollen, and bleeding upon the slightest touch. Os tincæ scarcely discernible for the tumefaction around. In the evening there was some increase of fever, and the patient complained of great soreness. I fear the introduction of the speculum has done harm. Ordered fifteen grains Dover's powder at bed-time.

21st.—Patient passed a very restless night. Intense pain over hypogastrium ; pulse 112, small and compressible ; thirst ; dry skin ; decubitus on the back, with legs drawn up. Ordered a dozen leeches to the back.

℞. Hyd. Chlorid mit. gr. xii.
Pulv. opii, gr. iv.
Tart. Emet. gr. iv.
M. Pil. 12—S. one pill every two hours.

22d.—No improvement. Bowels constipated ; pulse 120 ; feeble ; tongue dry ; complains of agonising pain along the sacrum ; some tympanites. Continue pills. Ordered an enema of soap-suds and castor-oil, which produced one evacuation, with comparative relief. Hot fomentations around the hips and hypogastrium.

23d.—Fever continues. Discharge from the vagina is *sanious* and fetid ; pain in the sacrum somewhat relieved. Added one grain of quinine to each of the pills, and gave them as before. Ordered f.℥xii. of magnes. citrat., to be taken at two draughts. Injections (vaginal) of pyroligneous acid, laudanum and water. Continue fomentations.

*Evening.*—Pulse 130, and feeble ; pain diminished ; tongue moist, and coated white; discharge from the vagina large and sanious, (I believe this to be the return of the catamenia;) bowels moved once; patient sleeps, and is sweating profusely; when aroused says she feels very weak. Continue treatment, with milk toddy.

24th.—Patient ptyalised; pain greatly diminished; pulse 100, small, but larger than yesterday; no appetite, but partakes freely of milk toddy and soup; sanious discharge still going on. Discontinue medicine.

25th.—All the symptoms better; passed urine to-day *sua sponte*, the first time since the operation; discharge from vagina less, but still sanious.

From this time *Jane* improved rapidly, so that on the 5th of November I was able to make a very thorough examination of the vagina. The lacerations had healed entirely, but there was so much contraction as barely to admit the forefinger up to the os tincæ; the constriction, however, was dilatable, and I introduced a sponge tent. In the course of a few hours this gave so much pain that it was removed by the nurse, but was again introduced on the next day, and borne with less inconvenience.

On the 12th November catamenia again returned, and continued until the 17th, the quantity and color being natural. During the flow, of course the tent was not worn, nor was any examination made. On the 20th I found that the contraction of the upper end of the vagina had increased; it would scarcely permit my forefinger to pass. The sponge tent was continued until she left the house on the 27th. We informed her master that she was not in a condition to go without treatment, and that unless the process of dilatation was continued, there would be a risk of the vagina becoming again closed. When she left the Infirmary, the forefinger, by exercising a moderate degree of perseverance, could be passed up to the os tincæ. The constriction yielding to persistent pressure like spasmodic stricture of the urethra.

On the 27th of April last Jane was again sent to the Infirmary. She stated that, until two months before, she had regularly had her monthly flow, although the discharge was less free, and accompanied with less pain than formerly. She had been under no treatment since I last saw her, the use of the tent being discontinued from the day she left the house. Upon examination I

found it impossible to reach the os tincæ, although there seemed to be an opening up to it. The speculum was introduced, and an attempt to find the opening made with a probe, but without success. No tumor could be felt from the rectum, nor fluctuation through the vagina, and yet I felt sure that there was menstrual blood locked up in the cavity of the uterus.

A second operation was contemplated, but it was deemed expedient to communicate with her master first. Pending the receipt of his reply, the patient was seized with a profuse discharge from the vagina of dark, grumous blood, more fetid than the pus from a rectal abscess. The discharge came on suddenly in the night, May 5th, and continued for five days, with entire relief to all the symptoms. Before any further examination was made a message was received from Dr. Southall, that her services were required at home, and that he would not have another operation performed yet. She was discharged on the 17th May, and I have not heard from her since.

*Remarks.*—The condition of the patient, when she last entered the Infirmary, is to be accounted for only upon the supposition that after the tents were discontinued, the vagina continued steadily to contract until it entirely closed, so far as the exit of a tolerably consistent fluid was concerned, while the admission of *air* might readily have been permitted. The contraction may have been of such a character as to make the small opening between the uterus and os externum, fistulous or *valvular* in its shape, thus locking up all secretion behind, but permitting a tolerably free ingress from without for the air, A certain amount of fluid having accumulated, an effort would be made to throw it off; this would produce irritation, possibly ulceration in the neighborhood of the constriction, and finally a breaking down of the obstruction, and a free exit to the fluid. There must have been an admission of atmospheric air to account for the excessive fetidness of the discharge.

It is probable that another operation will be required in this case. The first one may be considered, however, as entirely successful, and doubtless there would have been no ultimate difficulty had the process of dilatation been continued after she left the Infirmary the first time, or had she been permitted to remain until the treatment was completed.

The operation in this case is like to that performed in a similar one by Amussat, except that the *tearing* was completed at one operation, whereas in Amussat's case it required three or four *tearings*, at intervals of three days. As his case is one of considerable interest, and bears a close resemblance in many particulars to that just reported, I will give a brief history of it, condensed from the *Medico-Chirurg. Rev.* for October, 1836.

A girl, 15 years of age, was carried from Germany to Paris to consult some of the leading surgeons there. Her mother stated that she had enjoyed good health until about two years before, when she began to suffer severely from periodic attacks of pain in the abdomen, and in the region of the kidneys. At first they were attributed to obstruction of the bowels; but it was soon discovered that this was not the cause, and on examination it was found that there was no trace of any vagina.

When she came under Amussat's notice, in February, 1832, the abdomen was enormously distended; at the lower part was felt a firm, resisting, globular tumor, supposed to be the enlarged uterus. The external parts of generation appeared to be perfectly normal, but on separating the labia there was no vaginal orifice, but simply a concavity corresponding to the position of the orifice, and lined with a smooth mucous membrane. A catheter having, with some difficulty, been introduced into the bladder, it could be distinctly felt by a finger in the rectum, "in consequence of the great thickness of the intermediate parts;" and higher up the finger met with a large, rounded, tense, and fluctuating tumor, occupying the hollow of the pelvis. The nature of the case now became apparent. The urethra, bladder and rectum were tied together by cellular tissue, and the vagina, in at least two-thirds of its length, "mau quait absolument."

The prognosis was considered unfavorable. MM. Magendie and Marjolin thought the only thing that could be done was to perforate the uterus from the rectum, and thus relieve it from its contents. Boyer regarded the case as hopeless, founding his opinion on the history of all similar cases on record. He therefore strongly recommended that nothing should be done. Amussat, however, was unwilling to abandon the case as irremediable. He had but recently seen two cases of imperforation of the vulva, where he had succeeded in re-establishing the vaginal passage, by gradually tearing asunder the union between the urethra and rectum with blunt instruments and with the fingers. With the consent of the mother and daughter, he proceeded to try the same method in this instance. We used at first a large sized straight sound, pressing it with considerable force against the obstruction in the direction of the vagina "*comme pour faire un trou.*" Having continued this pressure for some moments, he found that there was quite a deep depression left, without laceration or loss of blood; a small piece of sponge was placed in the hollow thus made, and retained by a bandage. Three days afterwards the operation was repeated, with the fingers, the points being laid close to each other and then gradually separated so as to tear the tissues gently. By having a catheter in the urethra and a finger in the rectum the risk of wounding these organs was avoided. When two inches of the canal were gained, he could feel the distended uterus, and, on the 9th of March, he perforated the tumor with a trocar, widened the opening with a bistoury, and let off twelve ounces of dark blood.

In the course of three days, peritonitis set in, with great prostration, tympanites, and a great tendency to stupor. The case was considered very critical, but yielded at last to leeching and mercurialization. The patient ultimately recovered, and when seen by Amussat in July, 1834, two years after the operation, was in fine health, and menstruating regularly. The vagina was very contracted, but adhesion had been prevented by the frequent use of bougies. The true state of the parts, at that time, is described in the following words of Amussat, after consultation with Magendie and Marjolin: "il existait un petit vagin, qui s'etait formé aux depens des petites et meme des grandes levies, la muqueuse ayant eté attirée dans le vagin artificiel par la cicatrice; *et au fond de ce vagin on decouvrait un trou, ou fistule, qui conduisait á la matrice et par lequel l'ecoulement des regles se faisait librement.*" The part of this sentence italicised describes, I believe, the state of the parts in my patient, when she last left the Infirmary. Indeed the whole case bears a very strong resemblance to mine, not only in regard to its history and the condition of the genital organs, but also in regard to the mode of relief adopted.

The danger of fatal peritonitis after the operation for occlusion of the vagina, in cases of retained menses, is admitted by all surgical writers. Even when no violence is done during the operation to the surrounding tissues, the simple evacuation of the contents of the uterus seems to be inseparably connected with this formidable disease. In Amussat's case, the gentlest means were resorted to, and the whole operation conducted with all the caution and skill which distinguished that celebrated surgeon, and yet the patient barely escaped with her life. M. Capuron reported a fatal case in 1840, and was of opinion "that when any portion of the vagina is wanting, it is imprudent to have recourse to any operation, under the risk of compromising the life of the patient."

In the July No. of *Med. Chir. Rev.* for 1830, Mr. Worthington reports a case in which he "carefully divided with a scalpel a dense cellular structure, of about half an inch in thickness, situated at the orifice of the vagina, and gave exit to about a pound of dark colored fluid." Notwithstanding the abdominal tumor subsided immediately, and the girl felt greatly relieved, peritonitis set in three days after, and she died on the fourth day. Professor Languebec relates a similar case, in which death took place on the fifth day after the operation, and attributes the tendency to inflammation to the long retention of the menses.

Boyer, who has treated at considerable length of the various congenital defects of the female organs of generation, says, "death is the inevitable consequence of the accumulation of the mensus in the uterus, where there is a *complete* absence of the vagina." I presume, then, that such accumulation is proportionably fatal where there is *partial* absence of the vagina. He says, too, that after patients have escaped the wounding of the rectum and bladder during the operation, he "has known them die from inflammation of the uterus and of the adjoining parts."

But notwithstanding the dangers of the operation for retained menses, especially in those cases where there is total or partial absence of the vagina, no surgeon would be justified in refusing to perform it. Death must be the ultimate result if the accumulation is permitted to go on; this can but occur if an attempt is made to relieve by operation, and there is certainly *a chance* of success, of which the patient should always have the benefit.

Cases of congenital occlusion are fortunately very rare. I

speak not now of simple thickening of the hymen, which frequently serves to retain the menses, and is perforated with a thumb lancet, without difficulty or danger, but of that occlusion which results from a partial or total absence of the vagina. I can find but six cases reported since 1820, although I have carefully examined many of the leading Journals of England and the United States, for 30 years past. The operation, therefore, herein reported, has not been often performed, it is one of some interest, and for this reason I am induced to publish it.

*Norfolk, Va.*, July 16, 1853.

